# Artificial intelligence in estimating fractional flow reserve: a systematic literature review of techniques

**DOI:** 10.1186/s12872-023-03447-w

**Published:** 2023-08-18

**Authors:** Arefinia Farhad, Rabiei Reza, Hosseini Azamossadat, Ghaemian Ali, Roshanpoor Arash, Aria Mehrad, Khorrami Zahra

**Affiliations:** 1 Department of Health Information Technology and Management, School of Allied Medical Sciences, Shahid Beheshti University of Medical Sciences, Tehran, Iran; 2 Cardiovascular Research Center, Mazandaran University of Medical Sciences, Sari, Iran; 3 Department of Computer Science, Sama Technical and Vocational Training College, Tehran Branch (Tehran), Islamic Azad University (IAU), Tehran, Iran; 4 Department of Information Technology and Computer Engineering and Ophthalmic Epidemiology Research Center, Azarbaijan Shahid Madani University, Tabriz, Iran; 5 Research Institute for Ophthalmology and Vision Science, Shahid Beheshti University of Medical Sciences, Tabriz, Iran

**Keywords:** Machine learning, Fractional Flow Reverse, Functional evaluation

## Abstract

**Background:**

Fractional Flow Reserve (FFR) is the gold standard for the functional evaluation of coronary arteries, which is effective in selecting patients for revascularization, avoiding unnecessary procedures, and reducing treatment costs. However, its use is limited due to invasiveness, high cost, and complexity. Therefore, the non-invasive estimation of FFR using artificial intelligence (AI) methods is crucial.

**Objective:**

This study aimed to identify the AI techniques used for FFR estimation and to explore the features of the studies that applied AI techniques in FFR estimation.

**Methods:**

The present systematic review was conducted by searching five databases, PubMed, Scopus, Web of Science, IEEE, and Science Direct, based on the search strategy of each database.

**Results:**

Five hundred seventy-three articles were extracted, and by applying the inclusion and exclusion criteria, twenty-five were finally selected for review. The findings revealed that AI methods, including Machine Learning (ML) and Deep Learning (DL), have been used to estimate the FFR.

**Conclusion:**

This study shows that AI methods can be used non-invasively to estimate FFR, which can help physicians diagnose and treat coronary artery occlusion and provide significant clinical performance for patients.

## Introduction

Cardiovascular Diseases (CVD) are the most crucial cause of death worldwide [1]. These diseases have been among the most critical concerns in the last few decades [2], so approximately 18.5 million people died due to CVDs in 2019. Expectedly, the death rate due to this disease will increase by the year 2030 to reach 23.6 million [3]. Coronary Artery Disease (CAD) is the most common CVD, affecting more than twenty million adults in the United States and accounting for approximately one-third of deaths [4]. In this disease, plaque accumulation causes narrowing of the coronary arteries [5, 6], which can be partial or in the form of complete blockage of the coronary arteries and causes disruption of blood supply to the heart tissue [7]. Coronary artery narrowing or blockage leads to severe symptoms such as angina pectoris and even myocardial ischemia [8].

Based on the evidence, the functional severity of coronary artery stenosis is the leading cause of myocardial ischemia [9, 10]. Physiological evaluation is a determining factor for patients with CAD treatment decisions [11]. The Fractional Flow Reserve (FFR) method is used in the physiological evaluation. This method uses a pressure wire passing through the stenosis to measure the flow and blood pressure before and after the stenosis after injecting an agent such as adenosine [12].

FFR is the gold standard for the functional assessment of coronary arteries. Many pieces of evidence show that revascularization should be performed based on the functional assessment of the vessels [13–18]. Based on considerable clinical evidence, using FFR helps select the appropriate patients and lesions for treatment, avoids unnecessary procedures, reduces medical costs, and improves clinical outcomes [12]. However, despite the recommendations of treatment guidelines, the use of FFR for diagnosing CAD is minimal due to its complexity, high cost, and invasiveness [19]. Therefore, non-invasive methods of estimating FFR are of great interest.

In the last three decades, Artificial Intelligence (AI) has been widely used to improve the accuracy of diagnostic methods and decision-making based on CVD datasets [20]. As a subfield of AI, Machine Learning (ML) describes algorithms that analyze data logically, similar to how humans conclude [21]. Recently, AI techniques have been used to estimate FFR using Computed Tomography Angiography (CTA), X-ray Coronary Angiography (XCA), Optical Coherence Tomography (OCT), and Intravascular Ultrasound (IVUS) images. These methods have been highly regarded due to their non-invasive nature. To our knowledge, there has not been systematic studies reviewing the AI techniques in FFR estimation. Knowing that the AI techniques present different performance depending on the type of technques, type of features, and validadtion approaches, we aimed to to obtaing a better understanding of AI techniques in FFR estimation through findings answers to the following questions:

1. What AI methods have been used to estimate the FFR?
2. What imaging tools have been used to estimate the FFR?
3. How do AI techniques estimate the FFR?

## Methods

### Review methodology

#### Search strategy

The Preferred Reporting Items for Systematic Reviews and Metanalysis (PRISMA) [22] have been followed to perform this systematic review. The basic search string was ((“Deep Learning”) OR (“Machine Learning”) OR (“Artificial Intelligence”) OR (“Neural Network”) AND ((Fractional Flow Reserve)), and searches were performed without date constraint using IEEE Digital Library, Web of Science, PubMed, ScienceDirect, and Scopus databases. The search string syntax was adapted depending on the database requirements. The search was performed on the title, abstract, and keywords. Previously identified articles were also included in the process.

#### Eligibility criteria

This study analyzed only original articles published in English designed and developed AI methods to estimate the FFR.

#### Study selection

In the screening stage, three authors reviewed the articles based on their titles and abstract and removed the irrelevant articles. In the next step, the full text of the selected articles was evaluated by two researchers separately based on inclusion/exclusion criteria. Disagreements were resolved with the help of the third author through consensus and brainstorming.

#### Data extraction

In the data extraction stage, the type of imaging tool, number of patients, number and type of lesion, AI method, problem type, features used, feature extraction method, segmentation type, and model efficiency were extracted. The name of the first author, year, and place of publication of the article were also extracted. Finally, the obtained results were displayed in the form of structured tables (Table 1).

#### Critical review and quality assessment

The quality of studies was examined by two authors F.A and R.R, and disagreements were resolved by a third reviewer A.H. The quality of included studies was assessed based on the Quality Assessment for Diagnostic Accuracy Studies (QUADAS-2) tool to assess the risk of bias and applicability of primary diagnostic accuracy studies. This tool categorizes the risk of bias for individual studies as “low,” “medium,” or “high” [23].

**Fig. 1:**
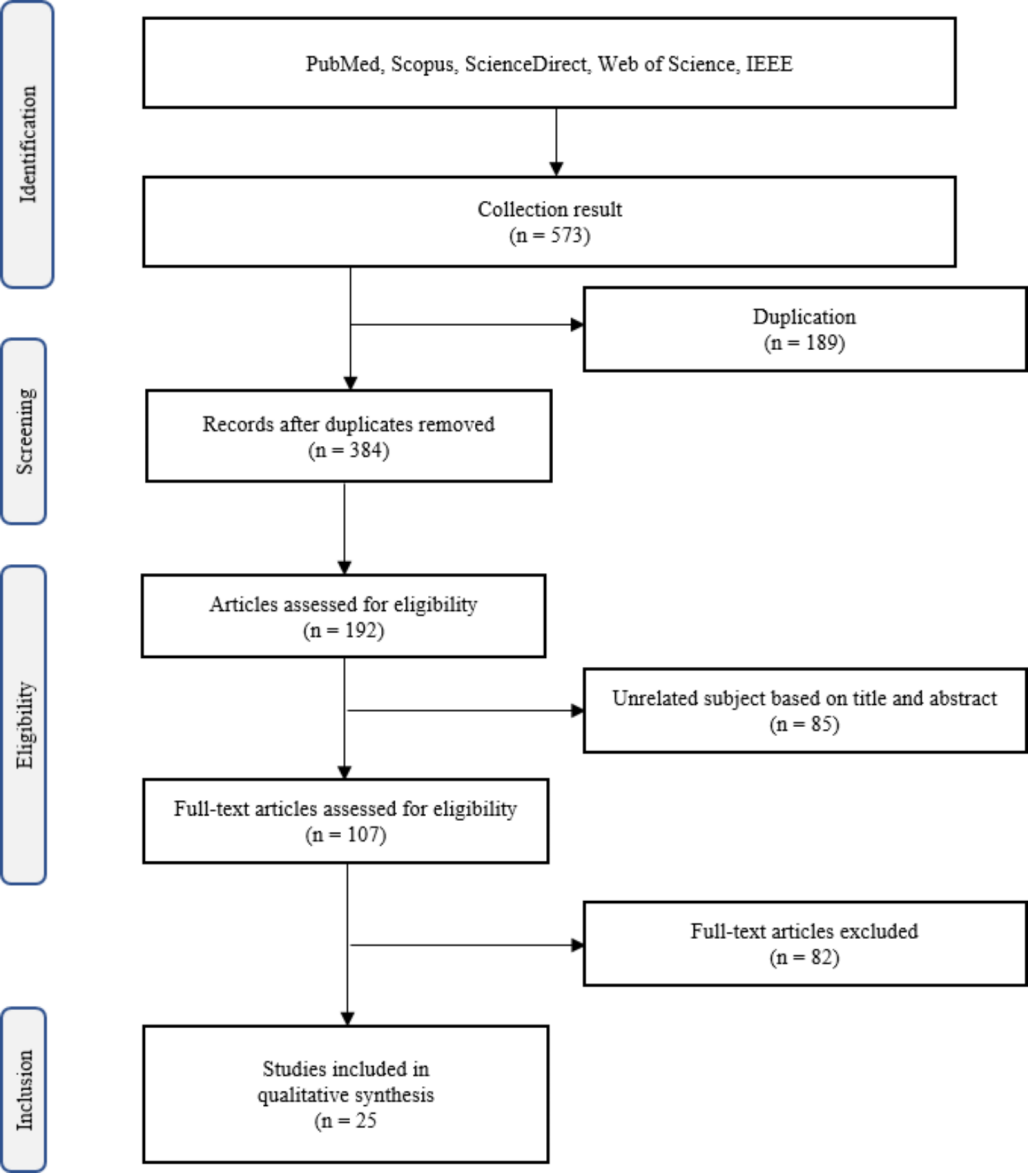
Flowchart of the study selection process (PRISMA)

## Results

The present study surveyed the title and abstracts of 384 articles. The full text of 107 articles was carefully examined, and eighty-two articles were excluded for reasons such as lack of full text, use of methods other than AI, conference articles, articles other than English, and articles with unclear results, and twenty-five articles were included in the study (Fig. 1).

The overall quality of most included studies was high. The subject selection method and follow and timing may have introduced high bias and applicability concerns in the reviewed studies.

This study demonstrated that various imaging tools have been used, including CCTA [24–40], OCT [41–43], XCA [44], and IVUS [45]. Several studies used a combination of CCTA with OCT [46], IVUS [47], and XCA [48], and a study combined IVUS and XCA [49] to estimate the FFR. Most of the studies used CCTA to estimate the FFR. AI methods used include methods based on DL [25, 27–29, 34, 40, 41, 48] and ML [26, 42, 30, 32, 33, 37, 39, 44, 46, 49]. Some studies have used a combination of DL and ML techniques [24, 32, 37, 38, 45, 47].

**Table 1:** Overview of FFR estimation studies included in this literature review

Reference (Year)	Modality	Number of patients/lesions	AI Methods	Prediction Task	Feature Engineering	Segmentation Task	Features	Performance	Quality assessment
Hatfaludi et al. (2022)[41]	OCT	80/102 (LAD = 57, LCX = 20, RCA = 25)	DNN	Classification	Feature learning (DNN)	A (Manually corrected by experts)	Anatomical OCT information	AUC = 0.763 Accuracy = 0.775 Sensitivity = 0.729 Specificity = 0.815 PPV = 0.778 NPV = 0.772	High
Xue et al. (2022)[48]	CCTA XCA	40/67(LAD = 32, D = 4, LCX = 10, OM = 1, RCA = 20)	BRNN	Regression	Feature learning (MLP)/ Handcrafted	M (DEEPVESSEL)/ A (U-Net)	Flow features Radius features Centerline Information	AUC = 0.95 Accuracy = 0.925 Sensitivity = 0.936 Specificity = 0.881 PPV = 0.8333 NPV = 1	High
Lee et al. (2021)[24]	CCTA	144/200(LAD) Synthetic	ANN, MLP RF, AdaBoost, SVM, GB, GP, KNN	Classification	Feature learning (InceptionV3)/ Handcrafted	A	Morphological feature Flow features Biometric features	Accuracy = 0.75 to 0.983	High
Roguin et al.(2021)[43]	XCA	31(LAD = 25, LCX = 3, RCA = 3)	ANN	Regression	Feature learning	A	-	Accuracy = 0.9 Sensitivity = 0.88 Specificity = 0.93 PPV = 0.94 NPV = 0.87	High
Fossan et al. (2021)[25]	CCTA	50(LAD = 26, LCX = 13, RCA = 11)/150 (LAD = 78, LCX = 39, RCA = 33)	FFNN	Classification	Handcrafted (VMTK)	M(ITK-SNAP)	Geometric features	Accuracy = 0.955 Sensitivity = 0.94 Specificity = 0.963	High
He et al.(2020)[26]	CCTA	60	SVM	Classification	Handcrafted (PyRadiomics)	M((Velocity)	left ventricular myocardial radiomics features	AUC = 0.8952 Accuracy = 0.855	High
Cha et al.(2020)[42]	OCT	125(LAD)	RF	Classification	Handcrafted	-	OCT Geometric feature Biometric features Clinical features	AUC = 0.98 Accuracy = 0.952 Sensitivity = 1 Specificity = 0.929 PPV = 0.875 NPV = 1	High
Kim et al., (2020)[46]	OCT CCTA	20	SVM	Classification	Handcrafted (Boruta)	-	Geometric feature Flow features Biometric features	Accuracy = 0.75 Sensitivity = 0.5 Specificity = 0.8 PPV = 0.83 NPV = 0.63	Moderate
Gao et al., (2020)[27]	CCTA	180/13,000 Synthetic	RNN	Regression	Feature learning (RNN)	A(U-Net)	Centerline Information	AUC = 0.93 Sensitivity = 0.84 Specificity = 0.89	Moderate
Carson et al. (2020)[28]	CCTA	25(LCA)	FFNN, LSTM, MPR	Regression	Handcrafted (VMTK)	M(VMTK)	Centerline Information	Accuracy = 0.72 Sensitivity = 0.9 Specificity = 0.6	High
Zreik et al.,(2019)[29]	CCTA	126/2340	CNN	Classification	Feature learning (CAE)	A(CNN)	LVM Computed features Centerline Information	AUC = 0.74 Accuracy = 0.7 Sensitivity = 0.7 Specificity = 0.7	High
YIN et al., (2019)[30]	CCTA	13(LAD)	GPR	Regression	Handcrafted	M	Physiologic parameters Anatomic parameters	Sensitivity = 0.76 to 0.91	Moderate
Dey et al., (2019)[31]	CCTA	254/484	LB	Classification	Handcrafted (AutoPlaque)	M (AutoPlaque)	Patient factors Quantitative CTA	Accuracy = 0.8 Sensitivity = 0.73 Specificity = 0.8	High
Zreik et al., (2019)[32]	CCTA	137/192(LAD = 104, LCX = 52, RCA = 36)	SVM	Classification	Feature learning (CAE)	-	Centerline Information	AUC = 0.87 Accuracy = 0.8	High
Lee et al., (2019)[45]	IVUS	1328/1328(LAD = 891, LCX = 100, RCA = 337)	RF, SVM, ANN, LR, AdaBoost, CatBoost	Classification	Handcrafted	A (VGG16- Manually corrected by experts)	Computed IVUS features Clinical variables Patient factors Quantitative CTA	Accuracy = 0.85 to 0.87	High
Kawasaki et al. (2020)[33]	CCTA	47/60	RF, LR, SVM	Classification	Handcrafted (CCTA Analysis)	-	Anatomic CCTA Descriptors Functional Descriptors	AUC = 0.698 to 0.835	High
Kumamaru et al. (2020)[34]	CCTA	1052 (**131 labelled** LAD = 118, LCX = 49, RCA = 40))	NN	Classification	Feature learning (cGAN^1^)	-	-	AUC = 0.78 Accuracy = 0.759 Sensitivity = 0.846 Specificity = 0.626 PPV = 0.777 NPV = 0.724	High
WANG et al. (2019)[35]	CCTA	63/71 (LAD = 32, LCX = 21, RCA = 18)	BRNN	Regression	Feature learning (MLNN^2^)	-	-	AUC = 0.664 Accuracy = 0.873 Sensitivity = 0.9714 Specificity = 0.75 PPV = 0.8293 NPV = 0.9545	High
Denzinger et al. (2019)[36]	CCTA	95/345	GRU	Classification	Feature learning (RCNN^3^) / Handcrafted (PyRadiomics)	-	Radiomic features Centerline Information	AUC = 0.88 Accuracy = 0.87 Sensitivity = 0.95 Specificity = 0.61 PPV = 0.9 NPV = 0.74	High
Cho et al. (2019)[44]	XCA	1501/1501(LAD = 1017, LCX = 155, RCA = 329)	XGBoost	Classification	Handcrafted (CAAS-5)	M(CAAS-5)	Computed angiographic features Clinical features	AUC = 0.87 Accuracy = 0.81 Sensitivity = 0.84 Specificity = 0.89 PPV = 0.77 NPV = 0.79	High
Hamersvelt et al. (2018)[37]	CCTA	126	SVM	Classification	Feature learning (CAE^4^)	A(CNN)	LVM Computed features	AUC = 0.76 Sensitivity = 0.846 Specificity = 0.484	High
Hae et al. (2018)[49]	XCA IVUS	1132/1132(LAD = 718, LCX = 141, RCA = 273)	RF, SVM, LR, AdaBoost, CatBoost	Classification	Handcrafted (CAAS-5/ EchoPlaque 3.0)	M (CAAS-5 for XCA and EchoPlaque 3.0 for IVUS)	Computed angiographic features Computed IVUS features Clinical features	AUC = 0.84 to 0.91 Accuracy = 0.78 to 0.84 Sensitivity = 0.76 to 0.84 Specificity = 0.8 to 0.85 PPV = 0.63 to 0.71 NPV = 0.88 to 0.92	High
Kim et al.,(2018)[47]	IVUS	70/ 1447	XGBensmble, ANN, XGBoost, RF	Classification	Feature learning (VGG16)	M /A ( VGG16- Manually corrected by experts)	Computed IVUS features Patient factors	Accuracy = 0.73 to 0.81 Recall = 0.63 to 0.71 Precision = 0.61 to 0.74 F1 score = 0.64 to 0.73	High
Zreik et al., (2017)[38]	CCTA	126	SVM	Classification	Feature learning (CAE)	A (CNN)	LVM Computed features	AUC = 0.74 Sensitivity = 0.71	High
Han et al.,(2017)[39]	CCTA	252/408	AdaBoost	Classification	Handcrafted (SmartHeart)	M (SmartHeart)	LVM Computed features	Accuracy = 0.683 Sensitivity = 0.527 Specificity = 0.846 PPV = 0.782 NPV = 0.63	High
Itu et al. (2016)[40]	CCTA	87/125 (12,000 Synthetic)	DNN	Classification	Feature learning	A (DNN)	Geometric features	AUC = 0.9 Accuracy = 0.832 Sensitivity = 0.816 Specificity = 0.839 PPV = 0.689 NPV = 0.912	High

^1^Conditional Generative Adversarial Network

^2^Multilevel Neural Network

^3^Recurrent Convolutional Neural Network

^4^Convolutional Auto-Encoder

In addition to the features extracted from the images, other features such as morphological, flow, biometric, clinical, radiomic, and centerline information have been used to estimate the FFR.

The extraction of parameters from imaging tools has been done manually, automatically, and in some cases with semi-automatic methods. Additionally, some of these studies used the segmentation technique to extract parameters from the images. This study shows that in some of these studies, DL methods were used for segmentation, and in others, the segmentation was done manually and using commercial software. Various parameters have been used to evaluate the diagnostic power of the models. The most used parameters are AUC, Accuracy, Sensitivity, Specificity, PPV, and NPV.

## Discussion

CADs are one of the severe complications in recent years, leading to myocardial ischemia. Numerous shreds of evidence show that the functional severity of coronary artery stenosis is the main reason for myocardial ischemia. The FFR is the gold standard for the physiological evaluation of coronary artery stenosis and for deciding on the revascularization of coronary artery stenosis. However, despite considerable clinical evidence, the use of this method is minimal due to limitations such as cost, complexity, and invasiveness. In this research, twenty-five studies have been systematically examined, and the findings are as follows:

## 1. AI methods

Various methods of AI, including methods based on DL and ML and a combination of them, have been used to estimate the FFR. A meta-analysis study needs to be conducted to evaluate these methods, which is practically impossible due to the variety of datasets.

## 2. Imaging tools

This study revealed that various imaging tools, including CCTA, XCA, IVUS, and OCT, were used. In addition, some studies have used a combination of imaging tools to estimate the FFR. The mentioned imaging tools are suitable for the anatomical assessment of coronary arteries. However, CHU et al., in a systematic review study, showed that by using the anatomical data extracted from these imaging tools, estimating FFR is possible [50]. This study’s findings specifically show the use of AI methods to estimate the FFR using different imaging tools, which can help the physician diagnose by aggregating anatomical and physiological parameters regardless of the type of imaging tool and treating the disease, which can significantly improve clinical performance for patients.

## 3. Type of vessels

Regarding frequency and type of vessels (Distribution of lesion types), angiographic interventions on the LAD branch are crucial [51]. This branch has the most CAD vulnerability, and the FFR is performed on it the most [25]. The present study findings also show that in most studies, the number of LAD branches is more than in other vessels, and since there is a better relationship between anatomical and functional parameters in this branch than in other branches, more studies are needed to generalize the results to other branches [52]. On the other hand, these vessels’ flow and anatomy differ [53]. Several studies show that the accuracy of predicting the FFR can be different according to the type of branches [54]. This study also shows that the results obtained separately for each vessel and each segment (proximal, mid, and distal) are different [25, 32, 41, 48]. Therefore, the separation of the type of vessels and the separation of each vessel according to the type of segment to determine the accuracy of the FFR estimation model is essential.

## 4. Features and Feature Engineering

Extracting quantitative imaging biomarkers using DL methods has two significant advantages. Firstly, they always return the same qualitative results from a specific input; secondly, like humans, there is no variance due to fatigue [55]. This study also shows that in several studies, image segmentation steps and feature extraction using DL methods have been done [27, 29, 32, 34, 35, 37, 38, 40, 41, 43]. In addition, in some studies, the parameters in the images were extracted using manual methods and commercial software [25, 28, 30, 42, 46, 30, 26, 45, 33, 44, 49, 39]. Due to the advantages of using automatic methods to extract the features of images, most studies have used automatic methods to extract features in the past year. According to the clinical guidelines of the American Society of Cardiology and the European Society of Cardiology, parameters such as age, sex, heart rate, blood pressure (BP), and past medical history are used to make decisions about ischemic heart disease [56, 57]. This study also shows that some of these studies have considered parameters such as age, gender, and clinical data to estimate the FFR. However, this study demonstrates that the effective parameters for estimating the FFR depend on the type of AI model used. For example, age and gender were essential parameters in the XGBoost model. However, they did not have much effect in RF [47], and the gender parameter in the model XGBoost was considered one of the critical parameters, but the age parameter was not influential [44]. In addition, in the RF model, age and gender are not important parameters, but BP is considered one of the influential parameters [42]. Numerous studies should evaluate these parameters with more data and different models to determine the effective parameters for determining the FFR.

## 5. Current challenges & future research

In recent years, end-to-end frameworks have been introduced in the field of DL, and the benefits of using them in health have been investigated [58, 59]. The present study shows that several studies used this framework to estimate FFR [27, 29, 32, 34, 35, 37, 38, 40, 41, 43]. Due to the need for the end-to-end framework for a large amount of data and the lack of data in these studies, the overfitting problem should also be considered [34], for which we need many data. Nevertheless, in this study, the number of patients in 85% of the studies is less than 250 people, which is a fundamental challenge because high-quality and large-volume data is needed in AI studies to achieve the desired result. Therefore, to solve this problem, some studies using accurate data have produced a synthetic coronary tree to train the model [27, 40]. In addition, the need for a dataset with labeled data and a large volume seems very necessary for studies of this kind. Another important challenge in these studies appears to be external validation, and it is suggested that researchers pay attention to it in future studies so that by accurate validation of these techniques, they could be applied in practice in FFR estimation. The importance of performing non-invasive FFR estimation with the aid of artificial intelligence techniques and the significant implications cannot be underestimated. In the future, cardiologists could benefit from the implications of AI in Estimation of FFR.

Eventually, the current study illustrates that AI methods for estimating the FFR have received the attention of researchers, and these methods are of great interest to cardiologists and patients due to their non-invasive nature and low cost.

### Limitations

In this study, the research for finding the revelvant literature was limited to studies published in English, and conference articles were not included in this study. In addition, the diversity of datasets used in different studies could impact the comparsion of different AI techniques in FFR estimation.

## Conclusion

This study reveals that various AI methods, including ML and DL and hybrid methods for predicting the FFR, have been designed and developed in recent years. These methods use different parameters, such as parameters extracted from different imaging tools for non-invasive estimation of FFR have been taken into consideration. There are a variety of imaging tools that have been used for predicting FFR, though these tools have limitations for physiological assessment. Studies suggest the significance of combining both anatomical and physiological parameters for diagnosing and treatment of the coronary disease in different stages of the disease. Due to the excellent performance of these methods, AI methods are an ideal, non-invasive, and cost-effective solution to solve the existing problem, which can bring good clinical performance for patients.

## Data Availability

The datasets used during the current study are available from the corresponding author on reasonable request.

## Abbreviations

A: Automatically
AI: Artificial Intelligence
ANN: Artificial neural network
BRNN: Bidirectional Multilayer Recursive Neural Network
BRNN: Bidirectional Multilayer Recursive Neural Network
CAD: Coronary Artery Disease
CCTA: Coronary Computed Tomography Angiography
cGAN: Conditional Generative Adversarial Network
CVD: Cardiovascular Diseases
DL: Deep Learning
DNN: Deep Neural Networks
FFR: Fractional Flow Reserve
GB: Gradient Boosting
GP: LogitBoost
GRU: Gated Recurrent Units
IVUS: Intravascular Ultrasound
LAD: Left Anterior Descending artery
LCA: Left Coronary Artery
LCX: Left Circumflex artery
LR: Logistic Regression
LVM: Left Ventricular Myocardial
M: Manually
ML: Machine Learning
MLNN: Multilevel Neural Network
MLP: Multilayer Perceptron
OCT: Optical Coherence Tomography
RCA: Right Coronary Artery
RCNN: Recurrent Convolutional Neural Network
RF: Random Forest
SVM: Support Vector Machine
XCA: X-ray Coronary Angiography
